# Supplementary material for: The genomic loci of specific human tRNA genes exhibit ageing-related DNA hypermethylation
Source: Nat Commun. 2021 May 11;12:2655. doi: 10.1038/s41467-021-22639-6 (PMC8113476; doi:10.1038/s41467-021-22639-6)
Supplement: Supplementary file 1 — Supplementary Information [file 41467_2021_22639_MOESM1_ESM.pdf]

Supplementary Information

Supplementary Figures

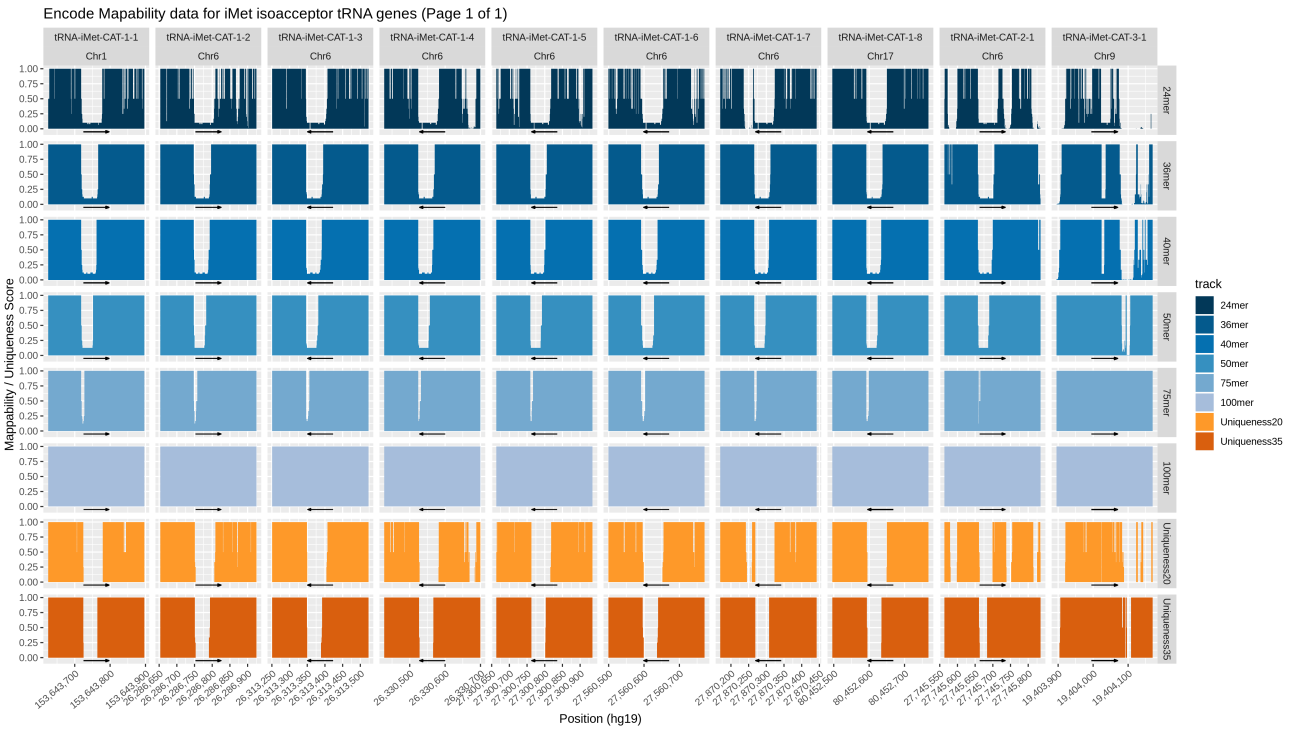

Supplementary Figure 1. Example of mappability data from the encode mappability tracks [1] for the initiator methionine tRNA genes. Source data are provided as a Source Data file.

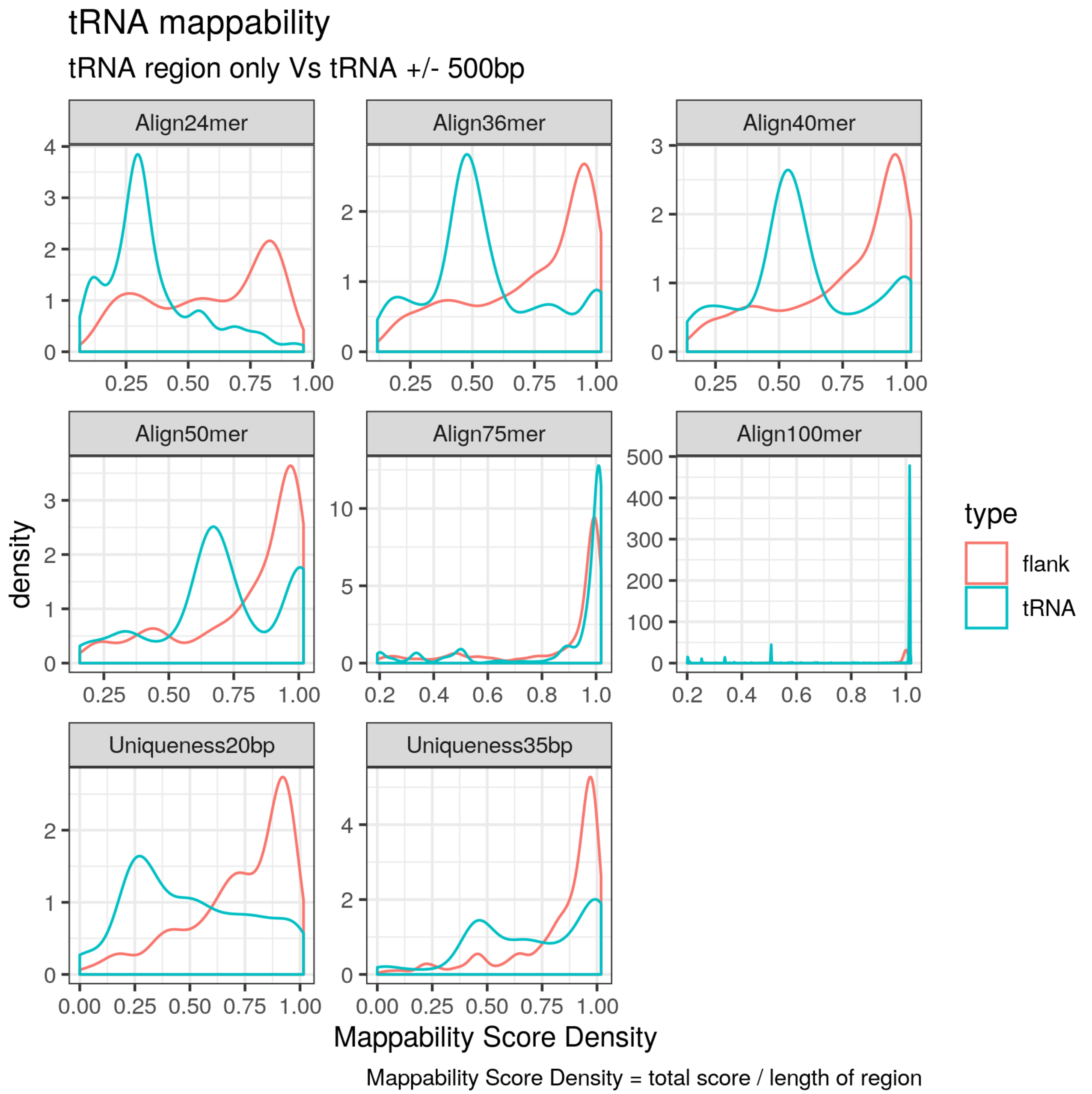

Supplementary Figure 2. Mappability score density of the tRNA genes increases with read length and is greater when flanking regions ($\pm 500bp$) are included. Mappability score density is computed as the area under the encode mappability tracks [1] over the length of the region. Source data are provided as a Source Data file.

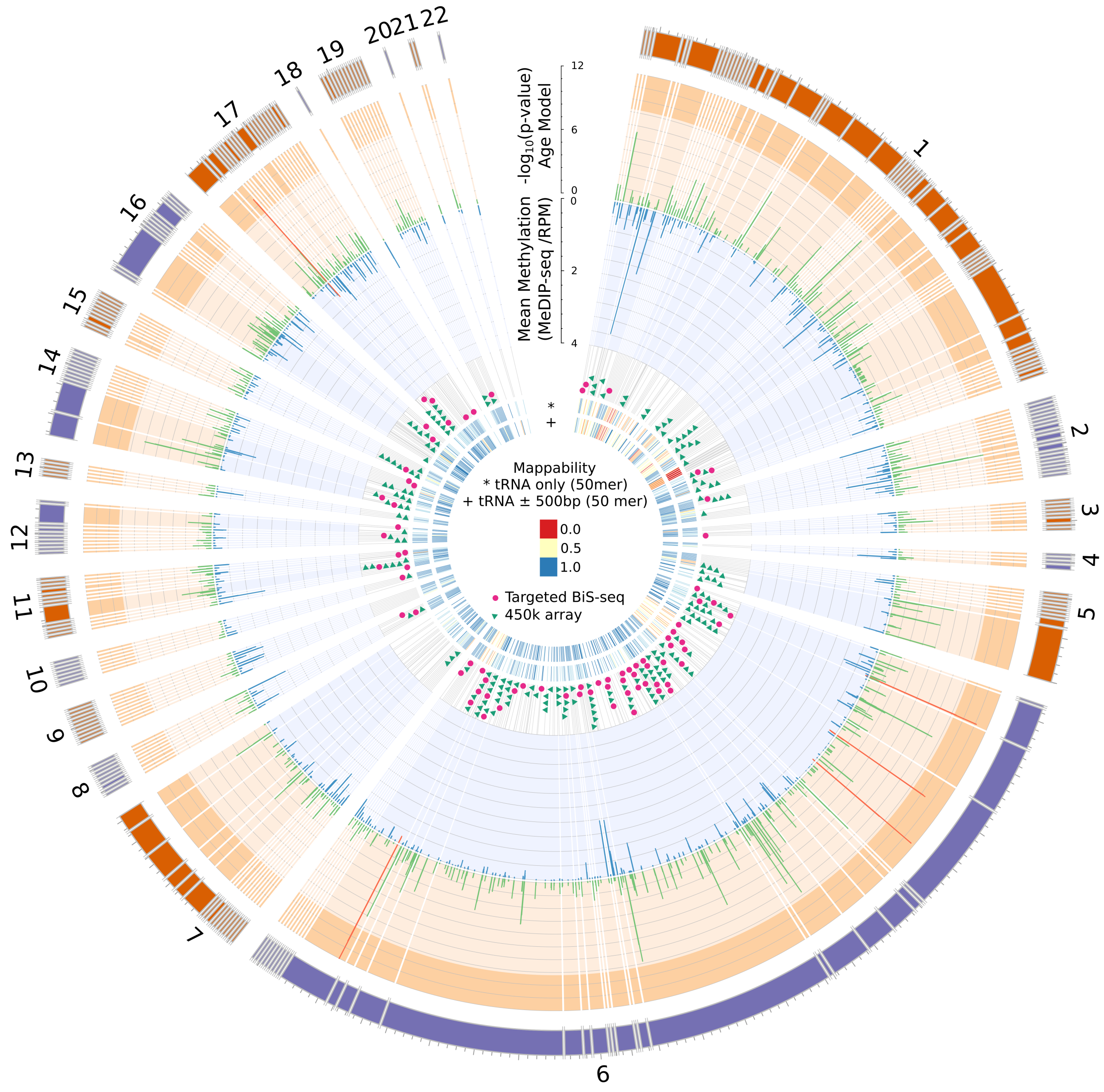

Supplementary Figure 3. Human tRNA genes overview. From the outside in: Chromosome ideograms scaled by the number of tRNA genes (total = 598), as excludes chromosome X (10), Y (0) and contig chr1_gl000192_random (2; see Methods). tRNA genes within 20kbp of one another are grouped with breaks inserted between these clusters. Radial grey lines represent the location of tRNA genes in the genome. $-log_{10}(p-value)$ for the blood cell-type and batch corrected age model are shown for each window overlapping a tRNA gene in green. Mean methylation across all samples (n=3001) in RPM (reads per million base pairs) is shown in blue. Genome-wide significant cell-type & batch corrected ($p < 4.34 \times 10^{-9}$) tRNAs show in red. The 158 Loci covered by 213 probes on the 450k array which directly overlap a tRNA gene are shown with green triangles. The 84 loci targeted for bisulfite sequencing in this study are indicated in magenta. Mappability score density is computed as the area under the encode mappability tracks [1] over the length of the region.

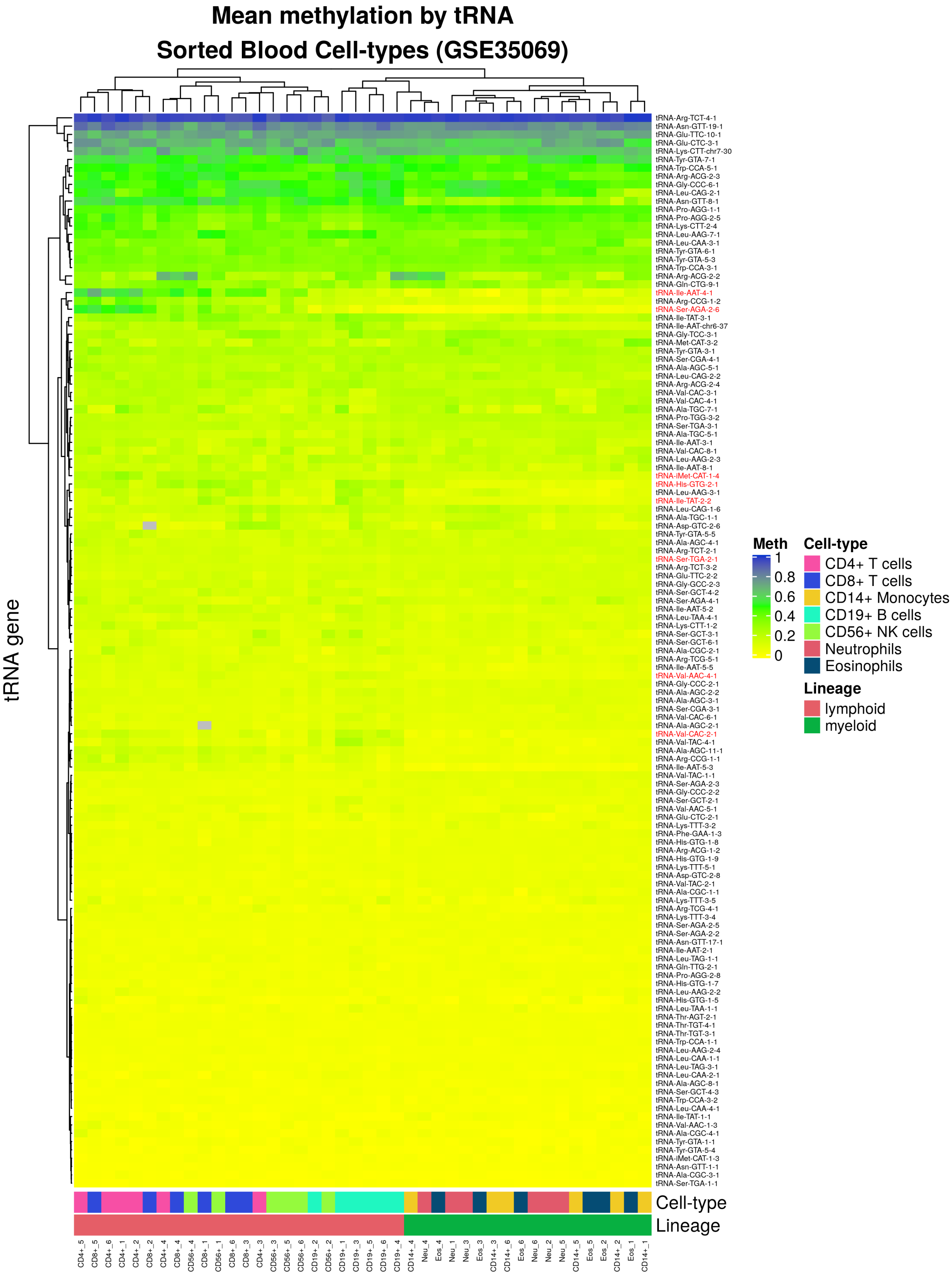

Supplementary Figure 4. Heatmap Mean Methylation of probes covering each tRNA in 7 cell-type fractions from 6 Male individuals. Showing all 150 tRNAs covered by 213 probes on the Illumina 450k array. Data from GSE35069 [2] downloaded using `GEOquery` [3,5]. Source data are provided as a Source Data file.

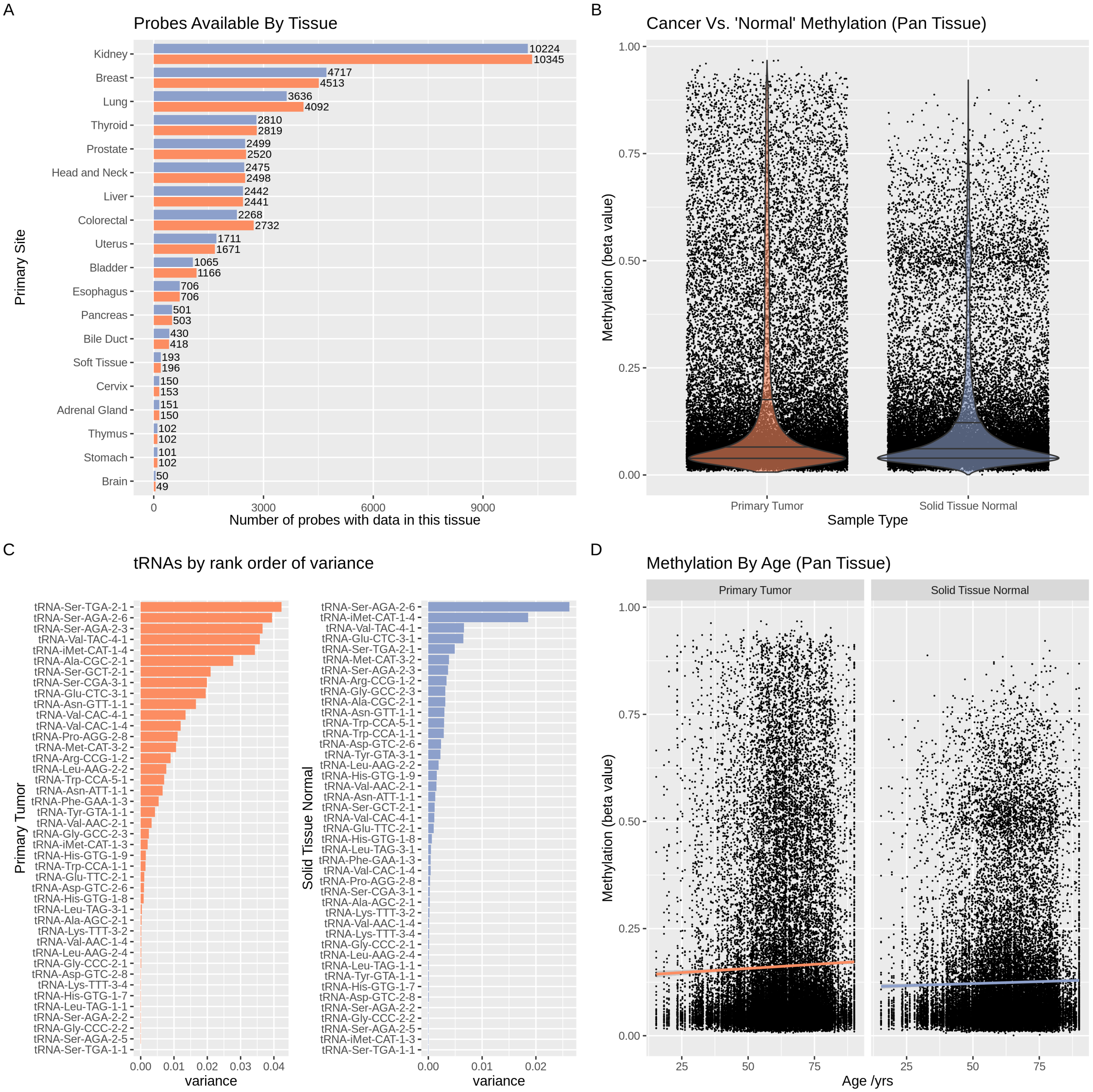

Supplementary Figure 5. Global properties of tRNA methylation data for 45 tRNA genes across 19 tissues with matched normal and tumour samples from 733 cases in TCGA [6,6]. The horizontal lines in the violin plots represent the 25%, 50% and 75% quantiles. a) Number of DNA methylation array probes with available data by primary site tissue type for normal tissue and primary tumour samples, b) DNA methylation level distribution for normal tissue and primary tumour site samples, c) Variance in DNA methyation levels by tRNA for normal tissue and primary tumour site samples, d) DNA methylation levels by age in normal tissue and primary tumour site samples, Source data are provided as a Source Data file.

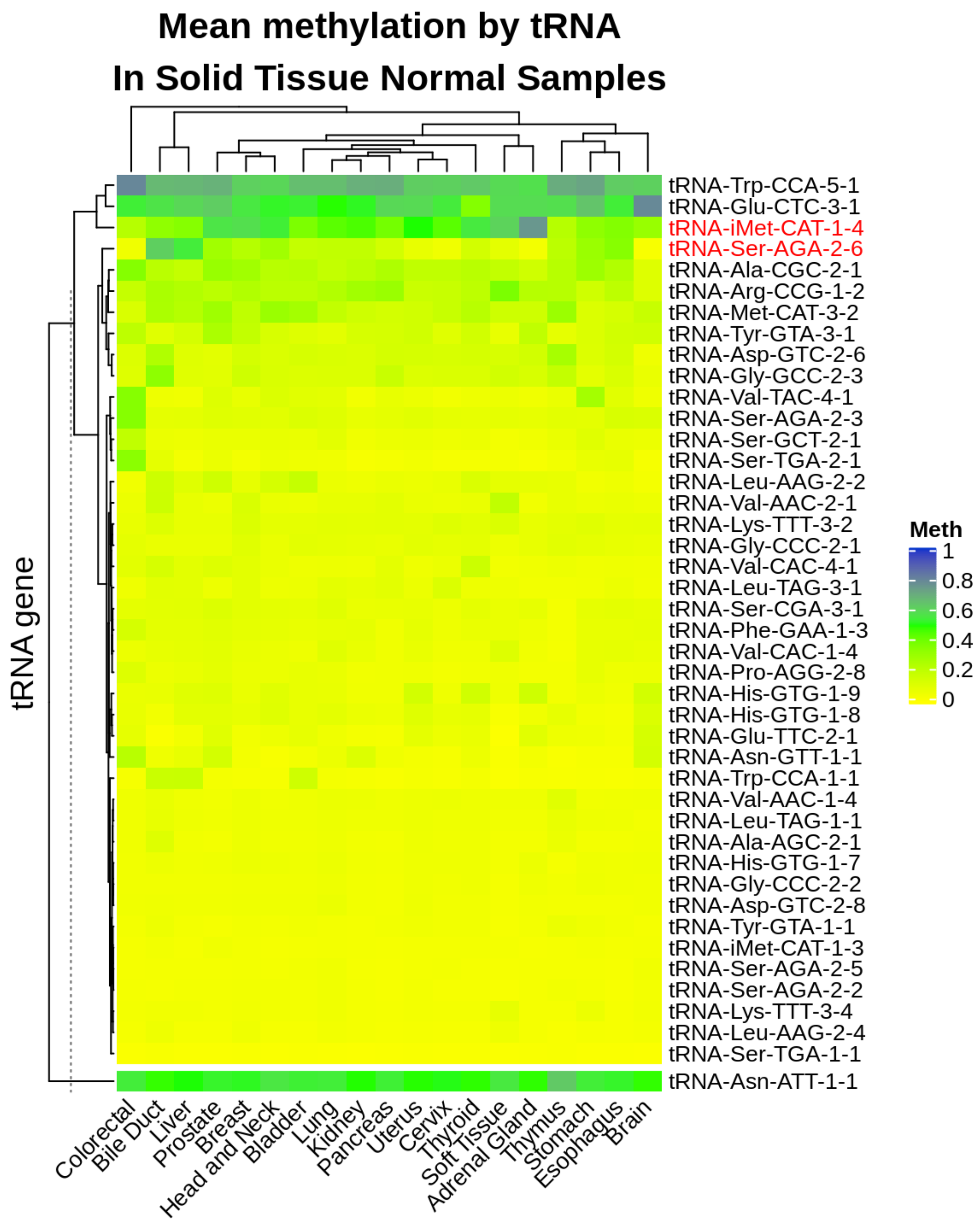

Supplementary Figure 6. Mean Methylation of 43 tRNAs in 19 tissues. Possible pseudogene (tRNA-Asn-ATT-1-1) is shown in a separate cluster beneath the main heatmap [3]. Source data are provided as a Source Data file.

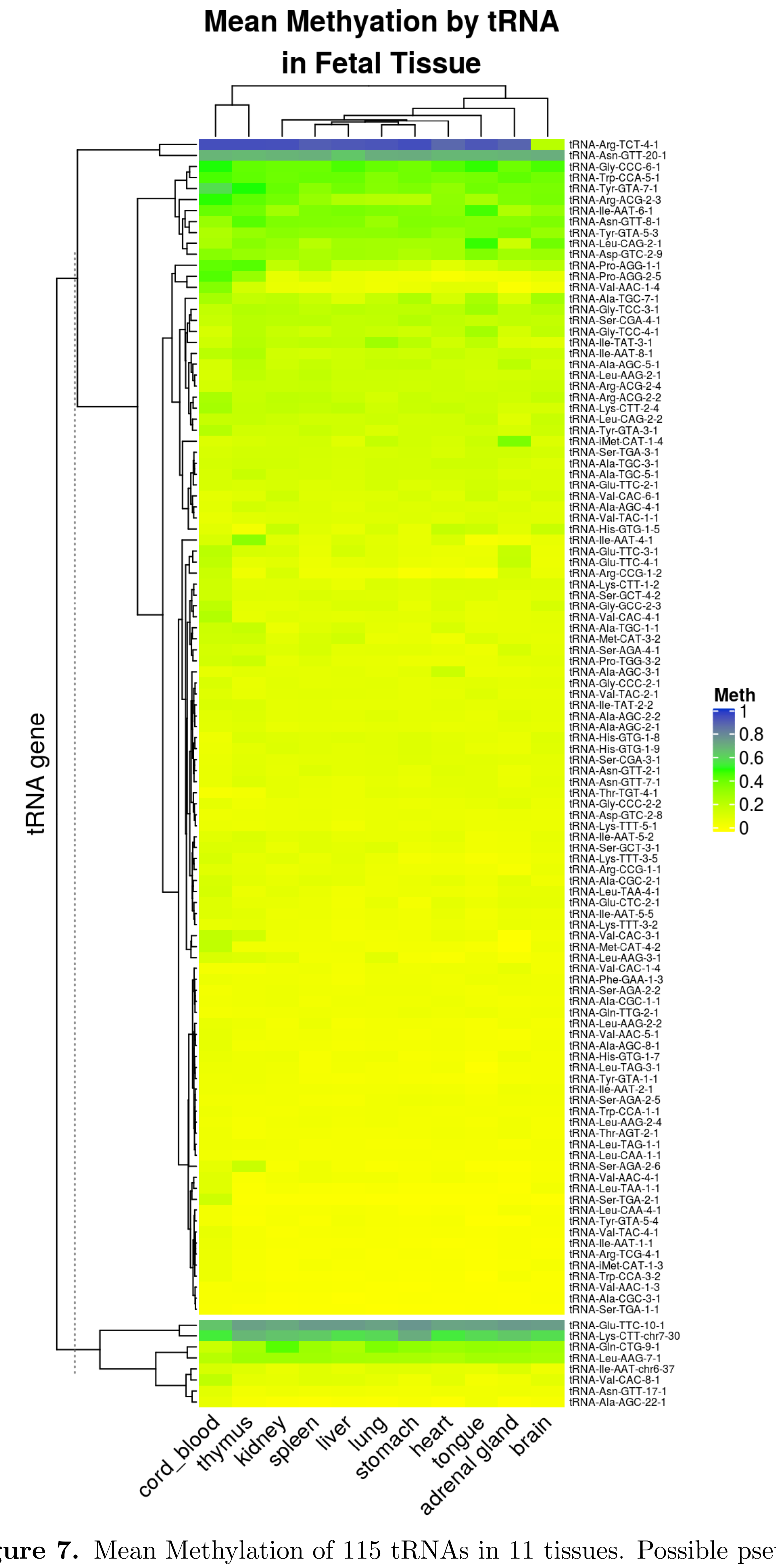

Supplementary Figure 7. Mean Methylation of 115 tRNAs in 11 tissues. Possible pseudogenes are shown in a separate cluster beneath the main heatmap [3]. Source data are provided as a Source Data file.

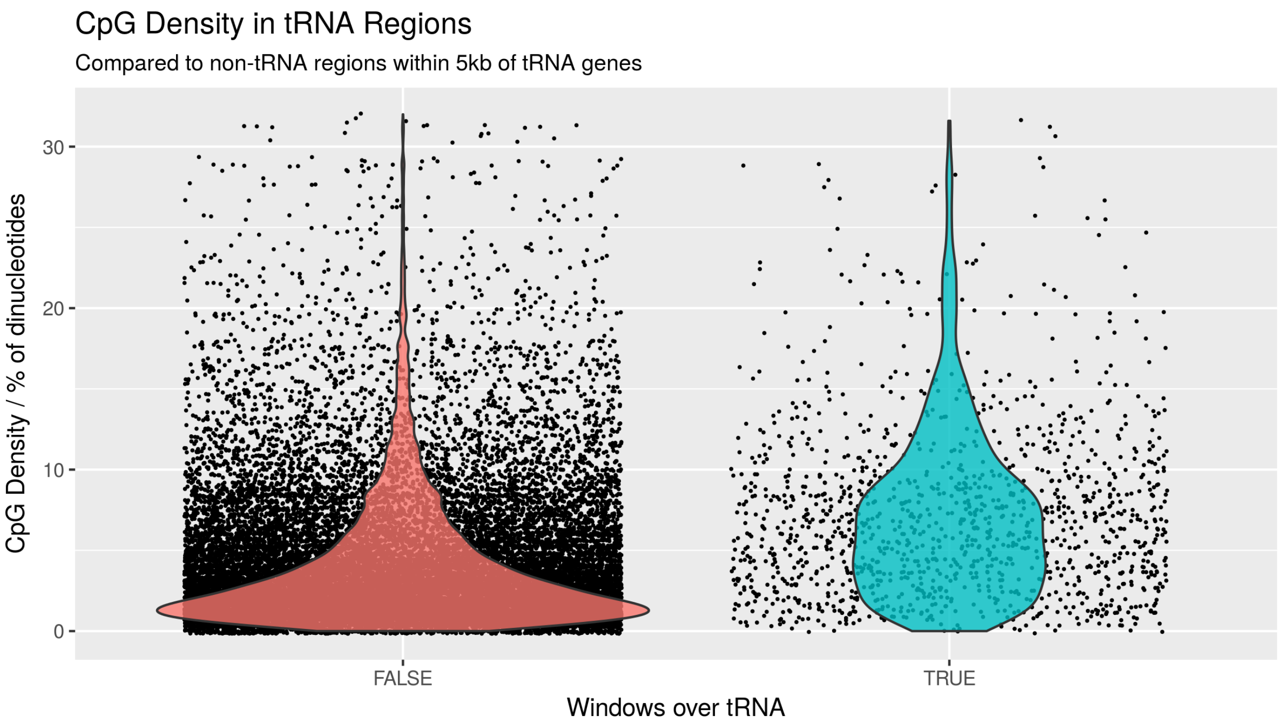

Supplementary Figure 8. CpG Density in windows overlapping tRNA genes compared to that of non-tRNA overlapping windows in flanking sequences (+/-5kb) Source data are provided as a Source Data file.

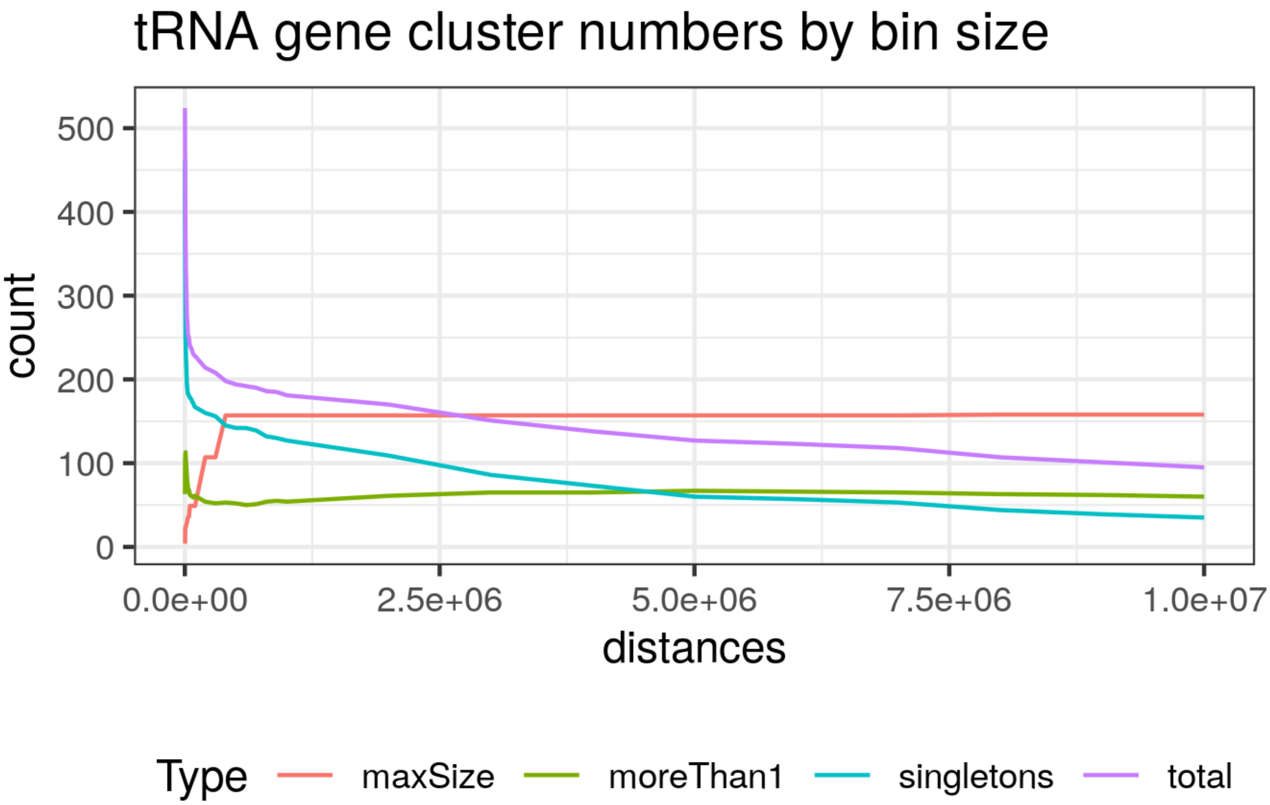

Supplementary Figure 9. tRNA gene cluster numbers at different bin sizes total: total number of tRNA clusters. singletons: number of tRNAs in clusters alone. moreThan1: number of tRNAs in clusters with more than one tRNA. maxSize: the number of tRNAs in the largest cluster. Source data are provided as a Source Data file.

Supplementary References

1.

Derrien T, Estellé J, Marco Sola S, Knowles DG, Raineri E, Guigó R, et al. Fast computation and applications of genome mappability. Ouzounis CA, editor. PLoS ONE. 2012;7: e30377. doi:10.1371/journal.pone.0030377

2.

Reinius LE, Acevedo N, Joerink M, Pershagen G, Dahlén S-E, Greco D, et al. Differential DNA methylation in purified human blood cells: Implications for cell lineage and studies on disease susceptibility. Ting AH, editor. PLoS ONE. 2012;7: e41361. doi:10.1371/journal.pone.0041361

3.

Gu Z, Eils R, Schlesner M. Complex heatmaps reveal patterns and correlations in multidimensional genomic data. Bioinformatics. 2016;32: 2847–2849. doi:10.1093/bioinformatics/btw313

4.

Nazor KL, Altun G, Lynch C, Tran H, Harness JV, Slavin I, et al. Recurrent variations in DNA methylation in human pluripotent stem cells and their differentiated derivatives. Cell Stem Cell. 2012;10: 620–634. doi:10.1016/j.stem.2012.02.013

5.

Sean D, Meltzer PS. GEOquery: A bridge between the gene expression omnibus (GEO) and BioConductor. Bioinformatics. 2007;23: 1846–1847. doi:10.1093/bioinformatics/btm254

6.

Yang Z, Wong A, Kuh D, Paul DS, Rakyan VK, Leslie RD, et al. Correlation of an epigenetic mitotic clock with cancer risk. Genome Biology. Genome Biology; 2016;17: 205. doi:10.1186/s13059-016-1064-3
