# Supplementary material for: The genomic loci of specific human tRNA genes exhibit ageing-related DNA hypermethylation
Source: Nat Commun. 2021 May 11;12:2655. doi: 10.1038/s41467-021-22639-6 (PMC8113476; doi:10.1038/s41467-021-22639-6)
Supplement: Supplementary file 3 — Description of Additional Supplementary Files [file 41467_2021_22639_MOESM3_ESM.pdf]

Description of Additional Supplementary Files

Supplementary Data 1: MeDIP-seq Age models tRNA windows. p-values are for F-tests from simple linear regression, and for anova for mixed models.

Supplementary Data 2: Targeted bisulfite sequencing age models by tRNA and by CpG. All p-values are for F-tests from simple linear regression.

Supplementary Data 3: TwinsUK 450k array age model results. All p-values are for F-tests from simple linear regression.

Supplementary Data 4: Mouse RRBS tRNA gene methylation age models. All p-values are for F-tests from simple linear regression.

Supplementary Data 5: tRNA coordinate annotation used in this work

Supplementary Data 6: Targeted Bisulfite sequencing primer sequences and target information

Supplementary Data 7: Details of the data files from TCGA/GDC used in the Cancer Vs. Normal and multi-tissue analyses.

Supplementary Data 8: TCGA data samples.
