# Supplementary material for: The genomic loci of specific human tRNA genes exhibit ageing-related DNA hypermethylation
Source: Nat Commun. 2021 May 11;12:2655. doi: 10.1038/s41467-021-22639-6 (PMC8113476; doi:10.1038/s41467-021-22639-6)
Supplement: Supplementary file 12 — Reporting Summary [file 41467_2021_22639_MOESM12_ESM.pdf]

nature research

Corresponding author(s): Christopher G. Bell (c.bell@qmul.ac.uk)
Richard J. Acton (r.j.acton@soton.ac.uk)

Last updated by author(s): Feb 5, 2021

Reporting Summary

Nature Research wishes to improve the reproducibility of the work that we publish. This form provides structure for consistency and transparency in reporting. For further information on Nature Research policies, see our Editorial Policies and the Editorial Policy Checklist.

Statistics

For all statistical analyses, confirm that the following items are present in the figure legend, table legend, main text, or Methods section.

n/a	Confirmed	
☐	☒	The exact sample size (*n*) for each experimental group/condition, given as a discrete number and unit of measurement
☐	☒	A statement on whether measurements were taken from distinct samples or whether the same sample was measured repeatedly
☐	☒	The statistical test(s) used AND whether they are one- or two-sided<br>*Only common tests should be described solely by name; describe more complex techniques in the Methods section.*
☐	☒	A description of all covariates tested
☐	☒	A description of any assumptions or corrections, such as tests of normality and adjustment for multiple comparisons
☐	☒	A full description of the statistical parameters including central tendency (e.g. means) or other basic estimates (e.g. regression coefficient) AND variation (e.g. standard deviation) or associated estimates of uncertainty (e.g. confidence intervals)
☐	☒	For null hypothesis testing, the test statistic (e.g. *F*, *t*, *r*) with confidence intervals, effect sizes, degrees of freedom and *P* value noted<br>*Give P values as exact values whenever suitable.*
☒	☐	For Bayesian analysis, information on the choice of priors and Markov chain Monte Carlo settings
☒	☐	For hierarchical and complex designs, identification of the appropriate level for tests and full reporting of outcomes
☐	☒	Estimates of effect sizes (e.g. Cohen's *d*, Pearson's *r*), indicating how they were calculated

Our web collection on statistics for biologists contains articles on many of the points above.

Software and code

Policy information about availability of computer code

Data collection

R version 3.5.2 packages:
- GEOquery v2.51.5
- GenomicDataCommons v1.6.0

Data analysis

Tools used:
R v3.5.2, key packages:
- tidyverse v1.2.1
- RnBeads v2.0.1
- limma v3.38.3
- ggplot2 v3.2.1
- ComplexHeatmap v1.20.0
- lme4 v1.1.21
MEDIPS v1.0
fastqc v0.11.5
multiqc 1.0
cutadapt 1.13
trim_galore 0.5.0
bowtie2 2.3.1
bedtools v 2.17.10
Bismark v0.2.0.0
tabix 1.3.2

Analysis code can be found here:
https://github.com/RichardJActon/tRNA_paper_code

For manuscripts utilizing custom algorithms or software that are central to the research but not yet described in published literature, software must be made available to editors and reviewers. We strongly encourage code deposition in a community repository (e.g. GitHub). See the Nature Research guidelines for submitting code & software for further information.

Data

Policy information about availability of data

All manuscripts must include a data availability statement. This statement should provide the following information, where applicable:
- Accession codes, unique identifiers, or web links for publicly available datasets
- A list of figures that have associated raw data
- A description of any restrictions on data availability

All data used in this study is publicly available under the following accession numbers.

Targeted Bisulfite sequencing data is deposited in the sequence read archive with the bioproject accession:
PRJNA635108 [https://www.ncbi.nlm.nih.gov/bioproject/PRJNA635108]

Neonate and Centenarian Whole Genome Bisulfite Sequencing DNA methylation calls:
GSE31263 [https://www.ncbi.nlm.nih.gov/geo/query/acc.cgi?acc=GSE31263].

Human isolated blood cell-type specific DNA methylation array Data:
GSE35069 [https://www.ncbi.nlm.nih.gov/geo/query/acc.cgi?acc=GSE35069].

Cancer and tissue specific DNA methylation array data from TCGA, see ([Supplementary Data S8](Supplementary_Files/SF8_TCGA_samples_used.tsv)) for the full list of samples drawn from genomic data commons. Feotal tissue DNA methylation data were downloaded from:
GSE72867 [https://www.ncbi.nlm.nih.gov/geo/query/acc.cgi?acc=GSE72867], &
GSE30654 [https://www.ncbi.nlm.nih.gov/geo/query/acc.cgi?acc=GSE30654].

Mouse whole blood RRBS DNA methylation data data:
GSE80672 [https://www.ncbi.nlm.nih.gov/geo/query/acc.cgi?acc=GSE80672].

The MeDIP-seq data supporting the results of this article are available in the EMBL-EBI European Genome-phenome Archive (EGA) under Data set Accession number EGAD00010000983 [https://www.ebi.ac.uk/ega/datasets/EGAD00010000983], access is subject to request and approval by their Data Access Committee.
The targeted BiS-sequencing data as a part of this work is available at: PRJNA635108 [https://www.ncbi.nlm.nih.gov/bioproject/PRJNA635108]
epilogos chromatin segmentation data is available from: [https://explore.altius.org/tabix/epilogos/hg19.15.Blood_T-cell.KL.gz]

Twins UK DNA metylation and age model summary data for non-overlapping 500bp windows is available at via UCSC Genome Browser's track hub interface, add:
http://epigenome.soton.ac.uk/TrackHub/hub.txt
Tracks include: mean, median and variance in RPM values across all samples in the model (n = 3001); the percentage of samples with an exactly 0 RPM score in a given window (useful for spotting technical issues); the slope and -log10(p-values) for batch corrected, and blood cell-type corrected age models.

tRNA gene annotations for the hg19 and mm10 genomes where acquired from GtRNAdb [http://gtrnadb.ucsc.edu/]

Source data for all figures are provided with this paper.

Field-specific reporting

Please select the one below that is the best fit for your research. If you are not sure, read the appropriate sections before making your selection.

☒ Life sciences ☐ Behavioural & social sciences ☐ Ecological, evolutionary & environmental sciences

For a reference copy of the document with all sections, see nature.com/documents/nr-reporting-summary-flat.pdf

Life sciences study design

All studies must disclose on these points even when the disclosure is negative.

Sample size	Our sample size of n=3001 samples in our blood cell-type corrected model is in excess of the n-2843 estimated to be required to detect very small effect sizes (R^2 = 0.02) at the genome-wide significance level of 4.34e-9 with 80% power
Data exclusions	In our targeted Bisulfite sequencing data we only used DNA methylation calls from loci with a minimum coverage threshold of 25 reads as this threshold substantially reduced the number of off target mapping reads when aligning to the whole genome. This cut-off threshold was not set prior to the acquisition of our data but was made before subsequent statistical analysis of DNA methylation levels at tRNA loci at different ages.
Replication	The specific result of Age-related DNA hypermethylation of tRNA-iMet-CAT-1-4 & tRNA-Ser-AGA-2-6 was identified in the Twins UK MeDIP-seq data, Validated in 450k array data on a subset of the same individuals present in the MeDIP-seq data and replicated in an independent cohort

	in the targeted bisulfite sequencing of pooled age-matched DNA. tRNA-Ile-AAT-4-1 Also initially identified as showing DNA hypermethylation with age in the MeDIP-seq data could not be reliably validated in the 450k array data due to an issue with the probe and failed to replicate in the independent cohort showing the opposite direction of change in this data.
Randomization	Randomization was not readily applicable to the analyses of cross-sectional data performed in this study. We were not asking questions about differences between groups but rather about changes over time with the populations available to us.
Blinding	Blinding was not readily applicable to our cross-sectional design as there were not randomized groups the state of which could be blinded

Reporting for specific materials, systems and methods

We require information from authors about some types of materials, experimental systems and methods used in many studies. Here, indicate whether each material, system or method listed is relevant to your study. If you are not sure if a list item applies to your research, read the appropriate section before selecting a response.

Materials & experimental systems

n/a	Involved in the study	
☐	☐	Antibodies
☒	☐	Eukaryotic cell lines
☒	☐	Palaeontology and archaeology
☒	☐	Animals and other organisms
☐	☒	Human research participants
☒	☐	Clinical data
☒	☐	Dual use research of concern

Methods

n/a	Involved in the study	
☒	☐	ChIP-seq
☒	☐	Flow cytometry
☒	☐	MRI-based neuroimaging

Antibodies

Antibodies used	MeDIP-seq used a monoclonal anti-5mC antibody to bind denatured fragmented genomic DNA at methylated CpG sites.<br>The kit used was the 'MagMeDIP' kit (Kit Cat. No.: CO2010021 mc-magme-048 from Diagenode (Liège, Belgium) https://www.diagenode.com/en/p/magmedip-kit-x48-48-rxns), and the monoclonal antibody was antibody 33D3 (C15200081 https://www.diagenode.com/en/p/5-mc-monoclonal-antibody-33d3-premium-100-ug-50-ul).<br>The antibody was incubated with Adaptor-ligated DNA combining 0.5µl antibody + 0.5µl water; then 0.6µl MagBuffer A, 1.4µl water and, 2µl MagBuffer C; yielding a final volume of 5µl for the immunoprecipitation reaction.
Validation	Validation information including for the use of this antibody in MeDIP is provided on the manufacturer's website in the datasheet for this antibody (https://www.diagenode.com/files/products/antibodies/Datasheet_5-mC33D3_C15200081-100.pdf)

Human research participants

Policy information about studies involving human research participants

Population characteristics	The MeDIP-seq dataset used in this work consists of 4350 whole blood methylomes with age data. 4054 are female and 270 male. 3001 have full blood counts. There are 3652 individuals in this data set. These individuals originate from 1933 unique families. There are 1234 monozygotic (MZ) twin pairs (2468 individuals), and 458 dizygotic (DZ) twin pairs (916 individuals). Age and sampling ranges from 16-18 years, median 56 years<br><br>The Characteristics of the Twins UK cohort are extensive documented here:<br>Moayyeri et al. 2013 https://doi.org/10.1017/thg.2012.89<br><br>Targeted Bisulfite Sequencing was carried out for 8 pools of DNA from individuals aged 4-80 years with 2 pools at time points for individuals at matched ages of 4, 28, 63 and 78 years. Supplementary file 3 contains additional details on the populations used in these pools. The MAVIDOS and Hertfordshire cohorts are extensively profiled in Syddall et al. 2005 DOI: 10.1016/S2213-8587(16)00044-9 and Cooper et al. 2016 http://dx.doi.org/10.1093/ije/dyi127 respectively.
Recruitment	Peripheral blood samples for DNA extraction were provided by the adult volunteers from the UK Twin Register details of the recruitment of these indicuduals can be found here: https://doi.org/10.1017/thg.2012.89
Ethics oversight	The Twins UK MeDIP-seq data received approval from the Guy's & St Thomas' NHS Foundation Trust Ethics Committee (EC04/015—15-Mar-04). MAVIDOS and Hertfordshire studies were overseen by the Southampton and South West Hampshire Research Ethics Committee and Hertfordshire and Bedfordshire Local Research Ethics Committees respectively.

Note that full information on the approval of the study protocol must also be provided in the manuscript.
